# Rondonin: antimicrobial properties and mechanism of action

**DOI:** 10.1002/2211-5463.13253

**Published:** 2021-08-19

**Authors:** Katie C. T. Riciluca, Ursula C. Oliveira, Ronaldo Z. Mendonça, José C. Bozelli Junior, Shirley Schreier, Pedro I. da Silva Junior

**Affiliations:** ^1^ Center of Toxins Immune‐Response and Cell Signaling – CeTICS/CEPID Laboratory for Applied Toxinology Butantan Institute São Paulo Brazil; ^2^ Post‐Graduation Program Interunits in Biotechnology USP/IPT/IBU São Paulo Brazil; ^3^ Laboratory of Parasitology Butantan Institute São Paulo Brazil; ^4^ Department of Biochemistry Institute of Chemistry University of São Paulo Brazil; ^5^ Department of Biochemistry and Biomedical Sciences Health Sciences Centre McMaster University Hamilton ON Canada

**Keywords:** antifungal peptide, antiviral, DNA binding, hemocyanin, rondonin

## Abstract

Infectious diseases are among the major causes of death in the human population. A wide variety of organisms produce antimicrobial peptides (AMPs) as part of their first line of defense. A peptide from *Acanthoscurria rondoniae* plasma, rondonin—with antifungal activity, a molecular mass of 1236 Da and primary sequence IIIQYEGHKH—was previously studied (UniProt accession number B3EWP8). It showed identity with the C terminus of subunit ‘D’ of the hemocyanin of the *Aphonopelma hentzi* spider. This result led us to propose a new pathway of the immune system of arachnids that suggests a new function to hemocyanin: production of antimicrobial peptides. Rondonin does not interact with model membranes and was able to bind to yeast nucleic acids but not bacteria. It was not cytotoxic against mammalian cells. The antifungal activity of rondonin is pH‐dependent and peaks at pH ˜ 4–5. The peptide presents synergism with gomesin (spider hemocyte antimicrobial peptide—UniProtKB—P82358) against human yeast pathogens, suggesting a new potential alternative treatment option. Antiviral activity was detected against RNA viruses, measles, H1N1, and encephalomyocarditis. This is the first report of an arthropod hemocyanin fragment with activity against human viruses. Currently, it is vital to invest in the search for natural and synthetic antimicrobial compounds that, above all, present alternative mechanisms of action to first‐choice antimicrobials.

## Graphical abstract

Rondonin's mechanism of action suggests yeast DNA/RNA binding, as it does not cause membrane disturbance. It acts synergistically with gomesin. It has superior action at pH 4 ˜ 5, and antiviral activity was detected against RNA viruses. This is the first report of an arthropod hemocyanin fragment with activity against human viruses.

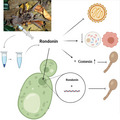

**Abbreviations**
AIDS: acquired immunodeficiency syndrome
AMP: antimicrobial peptide
ATCC: American type cell culture
DAPI: 4’,6‐diamidino‐2‐phenylindole
DEPC: diethyl pyrocarbonate
DMEN: Dulbecco's modified Eagle’s medium
DMSO: dimethyl sulfoxide
DNA: deoxyribonucleic acid
EDTA: ethylenediaminetetraacetic acid
FBS: fetal bovine serum
FICI: fractional inhibitory concentration indexes
FITC: fluorescein isothiocyanate
gDNA: genomic DNA
HIV: human immunodeficiency virus
LB: Luria–Bertani
LUV: large unilamellar vesicles
MIC: minimum inhibitory concentration
mRNA: messenger RNA
MTT: 3‐(4,5‐dimethylthiazol‐2‐yl)‐2,5‐diphenyltetrazolium bromide
PB: poor broth
PBSA: polymerized bovine serum albumin
PDB: potato dextrose broth
RNA: ribonucleic acid
SDS: sodium dodecyl sulfate
TFA: trifluoroacetic acid

## Introduction

Some fungal diseases began to receive attention in the last century, especially in the final two decades, with the advent of AIDS, advances in the treatment of basic diseases, greater use of antimicrobials, and improvement in transplantation techniques, that is, with the increasing patient survival. Among the fungi responsible for diseases in immunodepressed individuals and in apparently healthy individuals, species of the genus *Candida* are found [1, 2, 3]. The *Candida* genus consists of approximately 200 different species of yeasts, which normally live in the most diverse bodily niches, such as the oropharynx, oral cavity, skin folds, bronchial secretions, vagina, urine, and feces. Among the species that make up this genus, *Candida albicans* is more relevant due to its prevalence rate in normal and disease conditions [4, 5]. Yeasts of the genus *Candida*, in particular *C. albicans*, are opportunistic pathogens frequently isolated from the mucous surfaces of normal individuals [6]. They colonize the mucous membranes of all human beings during or shortly after birth, always with the risk of endogenous infection [7, 8, 9]. *Candida* species live as commensals, that is, being part of the normal microbiota of healthy individuals; therefore, when there is a disruption in the normal balance of the microbiota or the host's immune system is compromised, the species of the genus *Candida* manifest themselves in an aggressive, becoming pathogenic [1, 10] with the development of infections called candidiasis [11].

Candidiasis is a mycosis caused by yeasts of the genus *Candida*, in which the lesion can be mild, acute or chronic, superficial, or deep, and of a very variable clinical spectrum. The main causative agent of this disease is *Candida albicans* [12].

The normal vaginal microbiota is rich in peroxide‐producing lactobacilli (*Döderlein bacilli*), which form lactic acid from glycogen, present mainly in the cytoplasm of the squamous cells of the intermediate type of the vaginal epithelium, whose production is stimulated by female sex hormones. This mechanism provides adequate acidity in the vaginal environment (pH approximately 4.5), hindering the proliferation of most pathogens. Yeasts are an exception, as they proliferate in an acidic environment [13].

The vaginal flora composed of lactobacilli is an important defensive barrier to vulvovaginal candidiasis, and lactobacilli act on three different levels. First, they compete with fungi for nutrients. Second, they carry out a process of coaggregation, being able, in addition to competing with fungi, to block the epithelial receptors for them, inhibiting their adhesion to the vaginal epithelium. This defense mechanism is the most important. Third, they are capable of producing substances (bacteriocins) capable of inhibiting the germination of mycelia. This may explain why antibiotic treatments can trigger vulvovaginal candidiasis due to depletion of the vaginal flora [14].

The number of antifungals currently available for clinicians is limited, and the scenario is worsened by the rise of antifungal resistance to available drugs such as azoles, polyenes, and echinocandins [15, 16, 17]. Although these drugs target a marked fungal pathway, there is an urgent need to develop new antifungal agents that are more effective, are fungal‐specific, have no or reduced toxicity, and simultaneously do not cause resistance [18].

A recent 2020 WHO fact sheet (https://www.who.int/news‐room/fact‐sheets/detail/antibiotic‐resistance) has stated that antibiotic resistance has increased worldwide, reaching dangerous levels. New resistance mechanisms are appearing and spreading throughout the planet day by day, endangering health services’ ability to treat common infectious diseases. The antibiotic crisis has led to a pressing need for alternatives such as antimicrobial peptides (AMPs) [19]. AMPs are components of innate immunity, forming the first line of defense used by many organisms against invading pathogens through a simple mechanism but complex in function [20]. These peptides have emerged as central components of the innate defenses of both lower and higher organisms, and they are found in bacteria, fungi, plants, and animals [21, 22, 23, 24, 25].

The species richness and habitat diversity of invertebrates suggest that the absence of acquired immunity has in no way hindered their successes. The robust innate immunity of invertebrates holds great complexity and is analogous to the innate immunity of vertebrates [26, 27, 28]. Throughout evolution, the ability of an organism to protect itself from microbial or other species invasion has been a key factor for survival.

In some arthropods, hemocyanin is the major component of hemolymph, reaching 90%. In some species, it is present in concentrations exceeding 100 mg·mL^−1^ [29] and could act as a source of AMPs. Hemocyanin peptide fragments have been identified in shrimp *Penaeus stylirostris* and *Litopenaeus vannamei* [30], as well as in crayfish *Pacifastacus leniusculus* [31]. In the spider *Acanthoscurria rondoniae*, we identified an antifungal peptide named rondonin that has activity against yeasts, and its primary sequence was defined as IIIQYEGHKH, which comprises the C terminus of subunit ‘D’ of hemocyanin from *Aphonopelma hentzi* [32].

Most AMPs exert a direct microbicidal effect by disrupting the membrane integrity of the target organism and/or by translocating across the microbial membrane to reach the intracellular targets [33]. AMPs can interact with microorganisms by electrostatic forces between their positive amino acid residues and negative charges exposed on cell surfaces. In this sense, the sensitivity of prokaryotic and eukaryotic cells is directly related to the different physicochemical properties of the lipids found on both membranes [34, 35, 36]. Alternatively, it has been proposed that AMPs cause microbial death via interaction with intracellular targets by inhibiting cell wall, DNA, RNA, and/or protein synthesis [37, 38, 39]. Examples of AMPs with intracellular targets include teixobactin that prevents cell wall synthesis by binding the peptidoglycan precursor lipid II [40], indolicidin that binds to DNA thereby inhibiting DNA replication [41], the larvae peptide Lser‐PRP2 that is suggested to inhibit protein folding by binding to the bacterial chaperone DnaK [42], and the proline‐rich peptide Bac5 that blocks translation by binding to ribosomes [43].

Our group have been studying AMP, and these have already been characterized in different invertebrates, such as Acanthoscurrins and gomesin from the *Acanthoscurria gomesiana* and *A. rondoniae* spider [44, 45, 46], Lacrain from *Scolopendra viridicornis* [47], Pinipesin from *S*. *subspinipes subspinipes* [48], Longipin from the *Acutisoma longipes* [49], a fragment of a human fibrinogen peptide found in the hemolymph of *Triatoma infestans* [50], in blowfly larval excretions and secretions, Sarconesin and Sarconesin II from *Sarconesiopsis magellanica* [51, 52, 53], and venom from the spider *Loxoceles gaucho* [54]. All of these molecules are involved in the protection of these species and show how the discovery and characterization of bioactive peptides is very important, as well as having wide applicability, from the treatment of infectious diseases to cancer [55].

In this paper, the putative mechanism of action of rondonin, an antifungal peptide, as well as the possible therapeutic use against infections caused by yeast, using *C. albicans* as a principal biologic model is proposed.

## Materials and methods

### Peptide synthesis

Rondonin was synthesized by China Peptides Co., Ltd. (China) with 98% purity using the classic Fmoc methodology [56]. Gomesin was synthesized by the solid phase using the strategy t_Boc/BZL and/or Fmoc/t‐bu introduced by Merrifield [57].

### Microorganisms

*Escherichia coli* SBS363, *Micrococcus luteus* A270, *Candida albicans* MDM8, *Paecylomyces farinosus*, *Cryptococcus neoformans* VNI (WM148), *Saccharomyces* *cerevisiae* (industrial cepa) strains were obtained from the microorganism collection at the Laboratory for Applied Toxinology at Butantan Institute.

### Antimicrobial activity assays

The antifungal activity of rondonin was monitored by a liquid growth inhibition assay using the fungus *P. farinosus*, *C. neoformans* VNI (WM148), and *S*. *cerevisiae* (industrial cepa), and they were cultured in poor dextrose broth (½ PDB: 1.2 g potato dextrose in 100 mL of H_2_O at pH 5.0; 79 mOsM). The antifungal activity of rondonin was evaluated by using a modified protocol M‐27A2, according to the Clinical and Laboratory Standards Institute (CLSI). Antimicrobial activities were performed using a fivefold microtiter broth dilution assay in 96‐well sterile plates at a final volume of 100 µL. Mid‐log phase cultures were diluted to a final concentration of 1 × 10^4^ colony‐forming units per mL. The peptide was dissolved in sterile Milli‐Q water at a final concentration of 1 mm. The minimal inhibitory concentrations (MICs) for rondonin were based on the fivefold microtiter broth dilution assay. A serial dilution of the peptide in 96‐well sterile plates at a final volume of 100 µL was used. Twenty microliters of stock solution was applied to each well at serial dilution using a twofold microtiter broth dilution assay and added to 80 µL of the bacteria/yeast dilution. Microbial growth was measured by monitoring the increase in OD at 595 nm after incubation at 30 °C for 18 h (modified in ref. [58]). The MIC is defined as the minimal concentration of peptide that caused 100% growth inhibition [59]. Measurements were performed in triplicate.

#### pH‐dependent antimicrobial activity

The pH‐dependent antibacterial activities of rondonin were monitored by a liquid growth inhibition assay using the Gram‐negative bacteria *E. coli* SBS363 and Gram‐positive bacteria *M. luteus* A270 that were cultured in poor broth nutrient medium (PB: 1.0 g peptone in 100 mL of water containing 86 mm NaCl; pH 4.0 to 8.0; 217 mOsM). The yeast *C. albicans* MDM8 was cultured in poor dextrose broth (½ PDB: 1.2 g potato dextrose in 100 mL of H_2_O at pH 4.0 to pH 8.0; 79 mOsM).

#### Synergy evaluation in Checkboard assay

The peptides were dissolved in sterile ultrapure water at a final concentration of 1 mm (rondonin) and 6 μm of gomesin (stock solution). Poor dextrose broth was used (½ PDB: 1.2 g potato dextrose in 100 mL of H_2_O at pH 4.0; 79 mOsM). For further peptide dilution, 10 μL of only ½ PDB broth was added to the seven rows. Rondonin was serially diluted twofold across the columns, and gomesin was diluted in separate plates and then added to the test plate down the rows. The resulting checkerboard contained each combination of the two peptides in 6 doubly increasing concentrations, with wells containing the highest concentration of each antibiotic at opposite corners. Row A represents a positive growth control without drugs. All wells containing 20 μL of single peptides or a combination of peptides were inoculated with 80 μL ½ PDB medium of the yeast suspension (1 × 10^4^ colony‐forming units per mL). Column 12, containing 100 μL of ½ PDB medium, served as a sterility control. The plates were incubated at 30 °C. The MIC is defined as the lowest concentration of peptide that completely inhibits yeast growth. The synergistic interactions were expressed as the fractional inhibitory concentration index (FICI), which is calculated as the sum of MICs of the combination (MICc) divided by the MICs of the peptides alone (MICa): FICI = (rondonin MICc/rondonin MICa) + (gomesin MICc/gomesin MICa). The mean FICI was calculated from two independent experiments performed with the *C. albicans* MDM8 strain. A synergistic effect was defined at an FICI ≤ 0.5 and a nonsynergistic effect at an FICI between > 0.5 and ≤ 4 [60].

### Fluorescein isothiocyanate (FITC) conjugate preparation

Fluorescein isothiocyanate (FITC) isomer I (Sigma‐Aldrich^®^, St. Louis, MO, USA) was used following the protocol provided by Sigma‐Aldrich^®^. FITC was dissolved in anhydrous DMSO (dimethyl sulfoxide) at a concentration of 1 mg·mL^−1^ (light protected). Rondonin (2 mg·mL^−1^) was dissolved in 0.1 m sodium carbonate buffer pH = 9.0. For each 1 mL of peptide solution, 50 μL of FITC solution was added very slowly to 5 μL aliquots while gently and continuously stirring the peptide solution. After all the required amount of FITC solution was added, the reaction mixture was incubated in the dark for at least 8 h at 4 °C. Ammonium chloride (NH_4_Cl) was added to a final concentration of 50 mm and incubated for 2 h at 4 °C to quench the reaction. To purify FITC–rondonin, we acidified the mixture to a final concentration of 0.05% TFA.

The acidic supernatant was loaded onto classic Sep‐Pak C18 cartridges equilibrated in acidified water (0.05% TFA). After washing with acidified water, we submitted the samples to 0% and 80% acetonitrile acidified water (0.05% ACN/TFA). The 80% Sep‐Pak fraction was concentrated in a vacuum centrifuge, reconstituted in ultrapure acidified water, and directly subjected to C18 reverse phase on a semipreparative Júpiter C18 column equilibrated at room temperature with 0.05% TFA in ultrapure water. The sample was purified using acetonitrile/water/0.05% TFA gradients of 0–80% acetonitrile in 60 min at a flow rate of 1.5 mL·min^−1^. Ultraviolet absorbance was monitored at 225 nm. The eluted peak fractions were collected by hand and vacuum‐dried (SpeedVac Savant, Waltham, MA, USA), and the purity of the peptide was analyzed by mass spectrometry to confirm the coupling.

#### Fluorescence Microscopy

Measurements were performed using a fivefold microtiter broth dilution assay in 96‐well sterile plates at a final volume of 100 µL. Mid‐log phase cultures were diluted to a final concentration of 1 × 10^4^ colony‐forming units per mL. The peptide was dissolved in sterile ultrapure water at a final concentration of 1 mm, 80 μL of *C. albicans* cells (1 × 10^6^) diluted at poor PDB was deposited in each well, and 20 μL of FITC‐labeled peptides (final concentration 10 μg·mL^−1^) were added to the well. To confirm the killing effect of the peptides, *C. albicans* MDM8 cells were treated with peptide and Vectashield Mounting Medium for fluorescence with DAPI (4',6‐diamidino‐2‐phenylindole). Fluorescent images were acquired with an Olympus BX51 (Olympus, Tokyo, Japan) with a specific band‐pass filter to FITC and DAPI. Cell f software from olympus (v3.4) was used for image acquisition and analysis at the Laboratory of Cell Cycle at Butantan Institute.

### Gel retardation assay

#### DNA extraction

Aliquots of microorganisms (10^7^ cells) were maintained at −80 °C. Each aliquot was inoculated into 4 mL of rich media LB (Luria‐Bertani media) and grown for 20 h at 30 °C in a shaking incubator. Cells were transferred to another tube with 20 mL of LB media and incubated for 8 h in a shaker incubator. Cells were pelleted in a microcentrifuge tube at 20 000 ***g***, and the cell pellet was resuspended in 500 μL of lysis buffer (2% Triton X‐100, 1% SDS, 100 mm NaCl, 10 mm Tris/HCl (pH 8.0), 1 mm EDTA, and 50 μL SDS 10%). The tubes were placed in a dry ice‐ethanol bath for 5 min (until they were completely frozen) and then immersed in a 95 °C dry bath for 5 min to thaw quickly. The process was repeated once, and the tubes were vortexed vigorously for 30 s. Each aliquot was treated with 10 μL of proteinase K (10 mg·mL^−1^) (Sigma‐Aldrich, St. Louis, MO, USA) and maintained for 2 h at 60 °C in a dry bath. We added an equal volume of phenol and mixed well by inverting the tube until the phases were completely mixed. We centrifuged the samples at 4000 ***g*** for five minutes at room temperature. The upper aqueous phase was carefully transferred to a new tube, 250 μL of phenol and 250 μL of chloroform : isoamyl alcohol (24 : 1) were added, and the tube was centrifuged at 4000 ***g*** for five minutes. The upper aqueous phase was removed. The aqueous phase was transferred to a tube containing 500 μL of chloroform : isoamyl alcohol (24 : 1) and centrifuged at 4000 ***g*** for five minutes. We added 250 μL of 7.5 m ammonium acetate and 750 μL of 100% ethanol to the aqueous phase and precipitated it at −80 °C for 20 min. The samples were centrifuged at 10 000 ***g*** at 4 °C for 30 min. The DNA pellets were washed with 1 mL of ice‐cold 70% ethanol and centrifuged at 10 000 ***g*** at 4 °C for 15 min. The DNA pellets were air‐dried and resuspended in ultrapure water. RNA contamination was removed by adding 1 μL of RNase A (20 mg·mL^−1^) (Thermo Fisher Scientific, Waltham, MA, USA) and incubated for 30 min at 37 °C. The DNA was measured using ODS nanodrop checking by the ratio test.

#### RNA extraction

The protocol before was applied to grow the microorganisms used for RNA extraction. Cell pellets were maintained at −80 °C until use. We added 500 μL Buffer A (50 mm NaOAc, 10 mm EDTA, 1% SDS, and 1% DEPC). The tubes were vortexed vigorously for 30 s, and 600 μL phenol was added at 65 °C. The sample was maintained at 65 °C for 5 min and centrifuged at 14 000 ***g*** for 30 s. This step was repeated once. The aqueous phase was removed to another tube, 300 μL of phenol and 300 μL of chloroform were added, and the tube was centrifuged at 14 000 ***g*** for three minutes. The aqueous phase was removed, and 50 μL of 3 m NaOAc (pH 5.2) and 1 mL ice‐cold 100% ethanol were added and then precipitated at −20 °C for 15 min. The sample was centrifuged for 10 min at 14 000 ***g***. The supernatant was carefully discarded and dried for 10 min at room temperature. The RNA pellet was resuspended in 400 μL of ultrapure DEPC (diethyl pyrocarbonate)‐treated water, 40 μL of 3 m NaOAc, and 1 mL of ice‐cold 100% ethanol. The RNA was precipitated again at −20 °C for 15 min. The supernatant was discarded and dried at room temperature. Finally, the RNA pellet was washed with 1 mL 70% ethanol and centrifuged at 14 000 ***g*** for 5 min. The supernatant was discarded and air‐dried. The pellet was resuspended in ultrapure DEPC‐treated water and heated at 65 °C for 10 min to facilitate RNA solubilization. To obtain only RNA for the retardation assay, we used RNase‐free DNase I (supplied with MnCl_2_ (1 U·μL^−1^, Thermo Fisher Scientific)).

#### mRNA extraction

For mRNA extraction, 890 ng·μL^−1^ total RNA was used. The extraction was performed using the Dynabeads^®^ mRNA DIRECT ^TM^ Kit (Ambion, Life Technologies, Carlsbad, CA, USA) according to the manufacturer’s instructions.

#### DNA/RNA gel retardation assay

Yeast and bacterial DNA were purified according to the methods above. The genomic DNA (100 ng) or total RNA (89 ng·μL^−1^) was mixed with increasing amounts of peptide rondonin (1.2–12 μg). The mixtures (peptide + gDNA) were incubated at room temperature for 10 min and then subjected to gel electrophoresis on a 0.8% agarose gel in TAE buffer (40 mm Tris pH 8.3, 20 mm acetic acid, and 1 mm EDTA) [61, 62]. As negative control, yeast and bacterial DNA were incubated without rondonin.

#### mRNA integrity assay

The extracted mRNA (7.5 ng) was incubated with rondonin peptide (6 μg) at room temperature for 10 min and then subjected to an integrity analysis using an mRNA Pico Series II Bioanalyzer 2100 (Agilent Technologies, Santa Clara, CA, USA). We used rondonin and isolated mRNA as a control.

### Model membrane preparation

#### Large unilamellar vesicles (LUVs)

Phospholipids were acquired from Avanti Polar lipids (Alabaster, AL, USA) and used as received. Stock solutions were prepared in chloroform and quantified by measuring inorganic phosphorous according to Rouser *et al*. [63]. Lipid films of desired lipid mixtures (pure POPC and POPC : POPG 7 : 3 molar ratio) were prepared in glass tubes by solvent evaporation using a stream of nitrogen, and final traces of solvent were removed in a vacuum chamber for the least 2 h. The dried lipid films were resuspended in ammonium acetate 10 mm (pH 5) or sodium phosphate 10 mm (pH 7) by vigorous vortexing at room temperature. The lipid suspensions were submitted to five cycles of freeze‐thawing followed by thirty‐one passages through two stacked 100‐nm polycarbonate filters using an Avestin extruder at room temperature.

#### Fluorescence studies

Fluorescence spectra were acquired at room temperature in Hitachi F‐4500 equipment interfaced with a computer. The excitation wavelength was 275 nm, and the emission was scanned from 295 to 400 at 60 nm·min^−1^ with a photomultiplier at 700 V using 5‐nm excitation and emission slits. Spectra taken under the same conditions without peptide were subtracted from spectra of the samples, and dilution effects were corrected. The initial peptide concentration was 15 μm. The lipid concentration varied from 0 to 1.5 mm. Correction of scattering effects caused by LUV was performed by acquiring spectra of the amino acid Y under the same conditions according to ref. [64].

### Cytotoxicity

#### Cell cultures

VERO (African green monkey kidney, ATCC CCL‐81), MDCK (Madin–Darby canine kidney ATCC CCL 34), and L929 (NCTC clone 929; L cell, L929, derivative of Strain L—CCL‐1) cells were grown in plastic T‐flasks or on multiwell plates using Leibovitz‐15 (L15) medium containing 0.9 g·L^−1^ of D‐galactose and 0.3 g·L^−1^ of L‐glutamine and supplemented with 10% fetal bovine serum (FBS) incubated at 37 °C. HeLa (CCL‐2, adenocarcinoma—human cells, epithelial) and Y1 (CCL‐79™—*Mus musculus* epithelial—adrenal gland) cells were grown in DMEM supplemented with 1.2 g·L^−1^ NaHCO_3_, 25 mg·mL^−1^ ampicillin, 100 mg·mL^−1^ streptomycin sulfate, and 10% fetal bovine serum (FBS) and incubated at 37 °C and 5% CO_2_.

#### Cytotoxicity assay

The cytotoxic effects of rondonin were assessed by using a standard VERO cell assay and tumor cells HeLa and Y1. Briefly, exponential phase VERO cells (day 3) were exposed to different concentrations of rondonin (serial twofold dilution at initial concentration 200 μm) and incubated at 37 °C. The supernatant was removed, and the remaining living cells were verified after being fixed and stained with crystal violet (0.25% in 20% methanol). HeLa and Y1 cells were subjected to MTT assays. For the MTT assays, we used 96‐well sterile microplates where 80 μL of 10^4^ cells per well were plated and maintained for 24 h at 37 °C in a 5% CO_2_ atmosphere for adhesion and confluence of the cells. Then, 20 μL of the rondonin peptide (200 μm) was added in serial dilution to evaluate cytotoxicity against the two tumor cell lines. As a negative control for cytotoxicity, only PBSA and the positive control DMSO (20 μL) were used. Three wells were left blank for DMSO reading.

After incubation for 48 h at 37 °C in a 5% CO_2_ atmosphere, all wells were washed twice with PBSA and 100 μL of DMEM supplemented with 10% FBS and 0.5 mg·mL^−1^ MTT. After 4 h of incubation, the wells were photographed, the MTT solution was withdrawn, and 100% DMSO was added to all wells. The plate was stirred for 5 min. After stirring, the plate was held at rest for color stabilization. The reading was made at 595 nm in a Victor^3^ microplate reader (1420 Multilabel Counter/Victor3; Perkin Elmer).

## Results and Discussion

### Antifungal activity

To expand the spectrum of activities of the rondonin peptide, new liquid inhibition assays were performed. This peptide was shown to be active not only against yeasts of the genus *Candida* as previously seen [32]. The peptide was also shown to be active against the only encapsulated yeast capable of causing a disease to humans, *C. neoformans* [65]. This fungus has the characteristic of being opportunistic and can affect immunosuppressed patients [66]. The fungus has a polysaccharide capsule that protects it from phagocytosis, thus reducing the presentation of antigens by T cells, which causes a decrease in the host's immune response [67], which makes our results promising for the treatment against cryptococcosis.

No activity was observed against *S. cerevisae*, this yeast belongs to the phylum Ascomycota [], in which we can find the filamentous fungi *Aspergilus niger*, *Cladosporium* sp., and *Penicilum fumegatus*, which have been previously tested, and the peptide has shown no activity [32]. This yeast is associated with industrial processes such as bakery, ethanol, and wine production, as well as used in industry and pharmaceuticals to obtain Lepirudin [4] making the result found in this work very interesting.

The entomopathogenic fungus *P. farinosus* is found in tropical regions, being saprobic from forest soils and parasites of several species of arthropods. It was found in larvae of Lepidoptera, pupae of Diptera, Coleoptera, and Hymenoptera, and pupa of Hemiptera and Arachnida [69] (1990). Rondonin was not able to inhibit the growth of this fungus in the concentration tested against this pathogen as we can observe in the table below (Table 1).

**Table 1:** Antifungal activity of the rondonin peptide assessed by liquid growth inhibition assay. Evaluation of minimum inhibitory concentration of rondonin (maximum tested concentration 100 μm). The fungus was cultured in medium‐poor PDB (half potato dextrose broth), 30 °C for three days (ND—not detected in a tested concentration).

Microorganisms	Rondonin MIC (µm)	μg/100 μL
*Cryptococcus neoformans*	50–25	6.18–3.9
*Paecylomices farinosus*	nd	nd
*Saccharomyces cerevisae*	nd	nd

*Cryptococcus neoformans* is an encapsulated yeast‐like pathogen that causes meningoencephalitis in immunosuppressed individuals. Cryptococcal meningitis is one of the most important fungal infections developed in HIV‐1‐infected patients and the third most frequent in neurological complications in AIDS patients [70].

### pH‐dependent rondonin antifungal activity against *Candida albicans*

Initially, the bacteria *M. luteus* A270, *E. coli SBS 363*, and the yeast *C. albicans* MDM8 were tested in a wide pH range (pH 4, pH 5, pH 6, pH 7, and pH 8). PB broth for bacteria and PDB broth for yeast (see above in Section pH‐dependent antimicrobial activity). The growth inhibition assay was performed in 96‐well microtiters and after 18 h of incubation at 30 °C. *E*. *coli* SBS363 grown at pH 6–7, and *M. luteus* A270 grown at pH 7–8 but only *C. albicans* MDM8 grown in all pH broth were tested. In this case, we discarded the bacteria from this study. A previous study determined the minimum inhibitory concentration (MIC) of rondonin in the isolate *C. albicans* MDM8 [32]. To test the range of activity of rondonin, the peptide was submitted to a liquid growth inhibition test at MIC concentrations varying from pH 4 to 8. The results showed that rondonin presented activity at all pH values tested, and the best activity was at acidic pH (4 and 5) (Table 2).

**Table 2:** Antifungal activity of the rondonin peptide assessed by pH‐dependent liquid growth inhibition assay against the yeast *Candida albicans* MDM8. The poor culture medium PDB (half potato dextrose broth) was adjusted to different pH values (from 4 to 8) to evaluate the minimum inhibitory concentration range of the rondonin peptide at different pH values, with a maximum tested concentration of 100 μm.

Rondonin		Antifungal activity (*Candida albicans* MDM8 ‐ MIC)	
pH	Charge	μm	μg/100 μL
4.0	2.8	12.5–25	1.54–3.09
5.0	2.2	12.5–25	1.54–3.09
6.0	1.5	25–50	3.09–6.18
7.0	0.4	50–100	6.18–12.36
8.0	−0.5	50–100	6.18–12.36

Previous studies have shown that rondonin has an MIC in the range of 33.5–16.75 μm when tested against *C. albicans* MDM8, with its maximum tested concentration being 67 μm. According to its time of action, in death kinetics experiments, it was verified that in 10 min, there was no more viable blastoconidium [32].

Then, the optimal pH for rondonin was evaluated against three proposed microorganisms (*E*. *coli*, *M. luteus*, and *C. albicans*), but only the yeast *C. albicans* MDM8 grew at all pH tested, which varied from pH 4–8 according to the methodology written above. This yeast had homogeneous growth at all pH levels, thus making it possible to test the activity of the synthetic peptide according to the same methodology for the tests of growth inhibition in liquid medium. As shown in Table 1, the anticandidal activity of the rondonin was preserved at low pH, suggesting that these peptides could potentially be used to inhibit *C. albicans* infections in different compartments of the human body (e.g., the skin, the vagina, chronic dental foci) that have acidic pH environment [71].

Candidiasis is an infection caused by fungi from the genus *Candida* and can affect the skin, eyes, oral cavity, esophagus, gastrointestinal tract, vagina, and vascular system of humans. Most infections occur in patients who are immunocompromised or debilitated [72]. Vulvovaginal candidiasis is the most common form of mucosal disease, affecting up to 75% of women [73, 74]. In Brazil, candidiasis has become a public health problem. It is the 3rd leading cause of death from systemic mycosis in AIDS‐negative patients. Records indicate an increase in mortality from an annual average of 39 deaths between 1996 and 1998 to 54 between 2005 and 2006. Taking into account the deaths of AIDS patients with underlying cases of candidiasis, the disease is the 2nd leading cause of death from systemic mycosis, with 1780 deaths in Brazil from 1996 to 2006 [75]. Nosocomial candidiasis is also a public problem in Brazil [76].

Our results lead us to believe that rondonin may become a possible drug in the future. More experiments should be carried out, but these data demonstrate that in addition to being a potent antifungal, it acts in different concentrations at different pH values and does not have antibacterial activity; therefore, it may not harm the treatment, as it does not destroy the local bacterial flora.

### Rondonin Synergy Evaluation

The antifungal activity of antimicrobial peptide compounds was evaluated by using a modified protocol M‐27A2, according to the Clinical and Laboratory Standards Institute (CLSI) [77]. The minimum inhibitory concentration (MIC) of gomesin (*Acanthoscurria gomesiana* spider AMP [45]) was 0.6 μm, while the MIC of rondonin was 25 μm. In addition, growth inhibition of *C. albicans* MDM8 was observed with the combined treatment of 0.15 μm gomesin and 1.5 μm rondonin (Table 3).

**Table 3:** Antifungal activity of synthetic peptides rondonin and gomesin evaluated by the liquid inhibition assay (½PDB, pH 4) against the yeast *Candida albicans* MDM8 and the fractional inhibitory concentration index (FICI). The synthetic peptides rondonin and gomesin were evaluated alone to determine the MIC alone, with a maximum concentration tested for rondonin of 50 μm and gomesin of 1.2 μm. To evaluate the inhibitory fraction index, synthetic peptides were evaluated in combination in serial dilutions.

Peptide	*C. albicans* MDM8 (MIC)
Rondonin	25 μm
Gomesin	0.6 μm
Rondonin + gomesin	1.5/0.15

Furthermore, the fractional inhibitory concentration index (FICI) of the combination of gomesin and rondonin was 0.31. The mean FICI was calculated from two independent experiments performed with the *C. albicans* MDM8 strain. A synergistic effect was defined at an FICI ≤ 0.5 and a nonsynergistic effect at an FICI between > 0.5 and ≤ 4 [60].

This result indicated that these peptides act synergistically, suggesting that the first line of defense of the spider was likely in plasma, and after this, the degranulation of hemocytes liberated the antimicrobial peptides [44]. Among them, gomesin is also present on *A. rondoniae* hemocytes [46]

### Rondonin binding to model membranes

AMPs are generally small and cationic and acquire an amphipathic conformation after interaction with the cell membrane. Three models are generally considered to explain the permeabilization of the cell membrane [20, 37, 78, 79]: barrel‐stave [80], carpet [81], and toroidal pore [82]. Recently, it has been proposed that in some cases, cationic antimicrobial peptides may act by promoting the grouping of phospholipids negatively charged from bacterial membranes, in particular, the abundant lipid phosphatidylglycerol [83].

Biological membranes are highly organized systems that are essential for life and define a limit of separation between the cell and the external environment, thus maintaining cell integrity. In turn, it is the mediating element of communication between the cell and its external environment, constituting a highly selective barrier, which allows the creation of an intracellular compartment with its own chemical composition [84].

The most accepted model of biological membranes is the ‘fluid mosaic’ [85]. According to this model, lipids, for the most part, are organized in bilayers, forming a fluid matrix. However, with the progress of spectroscopic techniques used in the study of lipid bilayers, this model has undergone modifications to adjust them to experimental data [86].

Lipids are, by definition, a class of organic compounds of biological origin with very varied structures, characterized by their high solubility in organic solvents and low solubility in water [87, 88]. They usually have at least one alcohol group and an esterified fatty acid in their molecule.

To evaluate the binding of rondonin to model membranes, we took advantage of its intrinsic fluorescence, which is due to the presence of a Y residue in its primary structure [89]. Model membranes were prepared with zwitterionic POPC to mimic mammalian and yeast membranes and a mixture of POPC with anionic POPG (POPC : POPG 7 : 3 molar ratio) to mimic bacterial membranes [90, 91, 92, 93, 94].

**Fig. 1:**
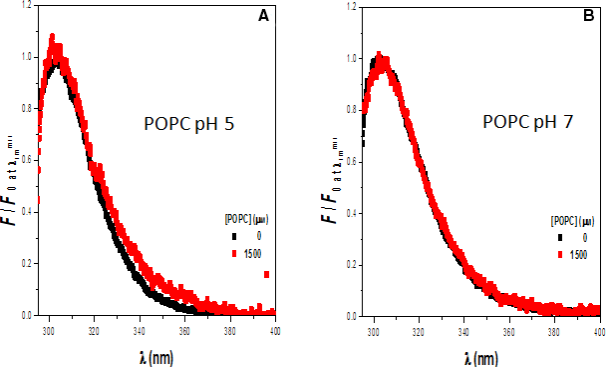
Interaction of the rondonin peptide with pure POPC model membranes at two pH values. Rondonin (15 μm) in the absence (black) and presence (red) of large unilamellar vesicles (LUV) composed of pure POPC. [Lipid] = 1.5 mm (*n* = 2).

Figure 1 presents the fluorescence spectra of rondonin in the absence and presence of pure POPC model membranes at pH 5 and 7. The fluorescence spectrum of Y is sensitive to the polarity of its microenvironment, which results in an increase in fluorescence intensity when the polarity decreases. The partition of the peptide from the aqueous phase to the membrane phase would lead to a decrease in the polarity of its microenvironment, and therefore, an increase in fluorescence intensity would be observed. The results showed that in buffer, the peptide presents a fluorescence spectrum characterized by a maximum at *ca*. 305 nm, which was similar in both pH values. In the presence of the model membranes, the spectrum essentially overlaps the spectrum in buffer at both pH values. These results suggest that rondonin does not interact strongly with these model membranes either at pH 5 or at pH 7. Since these model membranes were prepared to mimic yeast membranes, the data suggest that the mechanism of action of rondonin against yeast might not involve disruption of the microbial membrane; therefore, rondonin might exhibit its antifungal activity via an intracellular target mechanism.

**Fig. 2:**
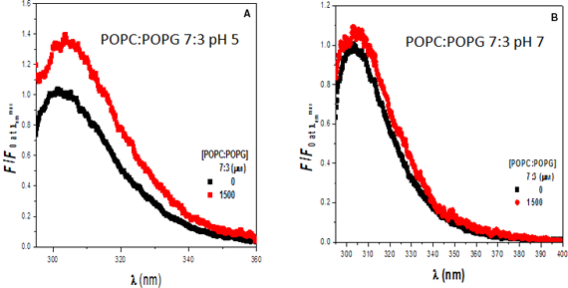
Interaction of the rondonin peptide with model membranes POPC : POPG 7 : 3 at two pH values. Rondonin (15 μm) in the absence (black) and the presence (red) of large unilamellar vesicles (LUV) composed of POPC : POPG (7 : 3, molar ratio) (*n* = 2).

Figure 2 presents the fluorescence spectra of rondonin in the absence and presence of model membranes composed of POPC : POPG 7 : 3 (molar ratio) at pH 5−7. At pH 7, the spectra of these model membranes essentially overlap, suggesting that the peptide does not interact strongly with these model membranes. On the other hand, at pH 5, an increase in fluorescence intensity is observed upon the addition of the model membranes, suggesting that the peptide interacts with the model membranes under this condition. These model membranes mimic the bacterial membrane, and the data indicate that the peptide–membrane interaction might play a role in the mechanism of action of this peptide against bacteria in acidic pH.

According to these results, it is hypothesized that rondonin exhibits its antifungal activity via a mechanism involving the interaction with an internal target [78, 95] as already described for other peptides [96], for example, indolicidin [97], mersacidin [98], buforin II [99], and tachyplesin [100]. Other examples are inhibition of 1,3‐β‐glucan synthesis that is involved in cell wall integrity [101, 102, 103] and inhibition of chitin biosynthesis in the cell wall that is essential to maintain cell integrity [104, 105].

### Cytotoxicity activity

Cytotoxicity tests of rondonin against mammalian cell cultures, as a prerequisite for performing the antiviral activity test, were conducted. A culture of VERO cells (renal cells of the African green monkey *Cercopithecus aethiops*), considered standard for these tests, was used (data not shown). The results showed that rondonin is not cytotoxic when incubated at an initial concentration of 200 μm starting with a serial dilution. Since the peptide is not cytotoxic, tests using human vaccine viruses could be evaluated.

To evaluate the antitumor activity of rondonin, cell cultures of cervical cancer (HeLa lineage) and cell cultures of mouse adrenal cancer (Y1) were used. The results showed that the peptide is not cytotoxic (Table 4) when evaluating cell viability by the MTT technique, which consists of the formation of the formazan crystal, which presents a violet color, from the metabolism of the MTT salt (3‐[4,5‐dimethylthiazol‐2‐yl]‐2,5‐diphenyltetrazolium) due to the cellular metabolic activity linked to NADH and NADPH. In this way, it is possible to evaluate that the amount of formazan, measured by spectrophotometry, is directly proportional to the number of viable cells [107].

**Table 4:** Evaluation of cell viability of HeLa and Y1 strains in the presence of the synthetic peptide rondonin by the MTT colorimetric method (3‐[4,5‐dimethylthiazol‐2‐yl]‐2,5‐diphenyltetrazolium bromide). The rondonin peptide, in serial dilution with a maximum concentration of 200 μm, was incubated in culture of two tumor cell lines, HeLa and Y1. After 48 h of incubation, cell viability was assessed by the MTT colorimetric method.

	Concentration (μm)	HeLa (%)	Y1 (%)
Rondonin	200	94.6	77.1
	100	87.9	80.9
	50	82.4	95.7
	25	84.6	97.3
	12.5	86.3	95.4
	6.25	90.6	90.8
	3.12	95.1	92.4
	1.5	71.6	93.7
DMSO	20%	0.8	3.6
PBS	20%	100	100

As described previously, VERO cells were exposed to different concentrations of rondonin using a fivefold microtiter broth dilution assay in 96‐well sterile plates at a final volume of 100 µL. Rondonin concentrations equal to or lower than 100 μm were not toxic to VERO cells, as high percentages of viable cells were observed. The results showed that rondonin, already characterized as an antifungal peptide against pathogenic, nonhemolytic fungal strains, does not interact with membranes and acts at acidic pH. Since it was not active against tumor cell lines, including cervical tumor cells (HeLa), it may become a possible candidate for a new drug against candidiasis, a disease caused mainly in women.

### Antiviral activity

The viral cytopathic effect (VCE) is defined as the set of changes caused by a virus in the cells where it multiplies. This effect can be manifested as changes in the shape and size of the cell, in the total destruction of the cell or in the detachment of the monolayer [106].

The antiviral action of rondonin was tested on 96‐well microplates containing a confluent cell culture.

**Fig. 3:**
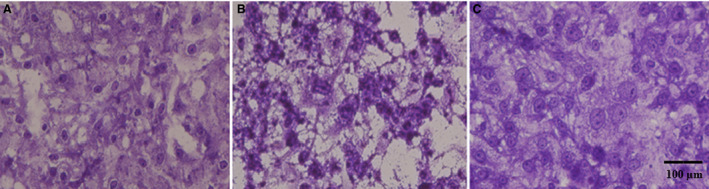
Determination of the antiviral activity of rondonin on MDCK cells infected with measles virus (Edmonston strain). Cells were treated with or without peptide and infected with MV. The plate was assessed daily by observing the appearance of a cytopathic effect. After 5 days, the cultures were stained with crystal violet. The plates were observed under an inverted optical microscope (x100). (A) negative control, only MDCK cells. (B) A positive control for infection, the cells infected with the virus. (C) Cells treated with the rondonin peptide similar to the negative control. The test was performed in triplicate (*n* = 3). Scale bar = 100 µm.

In Fig. 3, MDCK cells (Madin Darby canine kidney cells) were treated with rondonin at 9 μm 1 h prior to infection. After this time, the cells were infected with 0.01 MOI (ratio between virus copies in the infection divided by the number of cells to be infected—multiplicity of infection) of measles virus (Edmonston) at serial twofold dilution. Cells were observed daily for cytopathic effect (VCE) evaluation. After five days, the cells were stained with violet crystal for viewing in optical microscope. Rondonin was able to inhibit ½ dilution of the virus. The titer of the virus was given as 1/256 virus.

**Fig. 4:**
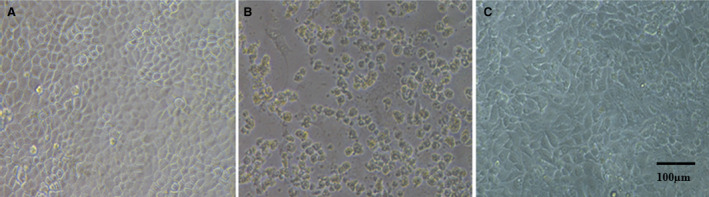
Determination of the antiviral activity of rondonin on Vero cells infected with influenza virus (H1N1). Cells were treated with or without peptide and infected with H1N1. The plate was assessed daily by observing the appearance of a cytopathic effect. After 3 days, the cultures were stained with crystal violet. The plates were observed under an inverted optical microscope (x100). (A) Negative control, only VERO cells. (B) A positive control for infection, the cells infected with the virus. (C) the cells treated with the rondonin peptide were similar to the negative control. The test was performed in triplicate (*n* = 3). Scale bar = 100 µm.

In VERO cells, as seen in Fig. 4, treatment with 24 μm rondonin was administered 1 h before infection. After this time, the cells were infected with 0.01 MOI of influenza virus (H1N1). Cells were observed daily for cytopathic effect (VCE) evaluation. After three days, the cells were stained with violet crystal for viewing in optical microscope. Rondonin was able to inhibit the 1/64 dilution of the virus. The titer of the virus was given as 1/256 virus.

**Fig. 5:**
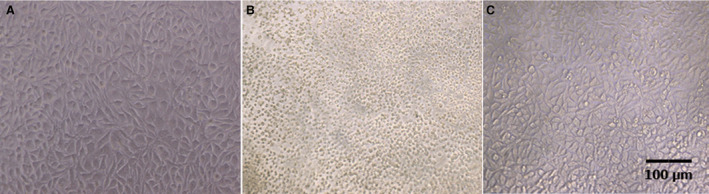
Determination of the antiviral activity of rondonin on L929 cells infected with EMC virus (Encephalomyocarditis virus ). Cells were treated with or without peptide and infected with ECM virus. The plate was assessed daily by observing the appearance of a cytopathic effect. After 3 days, the cultures were stained with crystal violet infected with EMC virus (encephalomyocarditis virus). The plates were observed under an inverted optical microscope (x100). (A) negative control, only L929 cells. (B) A positive control for infection, the cells infected with the virus. (C) Cells treated with the rondonin peptide similar to the negative control. The test was performed at triplicate (*n* = 3). Scale bar = 100 µm.

In L929 cells (mouse fibroblast cells), as shown in Fig. 5, the cells were treated with 9 μm rondonin peptide 1 h before infection. After this time, the cells were infected with 0.01 MOI of EMC viruses. Cells were observed daily for cytopathic effect (VCP) evaluation. After three days, the cells were stained with violet crystal for viewing in optical microscope. Rondonin was able to inhibit the highest concentration of the virus. The titer of the virus was given as 1/4096 virus.

Hemocyanins are a new class of natural antiviral compounds against different viruses. Therefore, hemocyanins of arthropods are active only against arthropod‐specific viruses such as white spot syndrome virus (WSSV) and Taura syndrome virus (TSV), while mollusks’ hemocyanins are active against human viruses, such as ‘respiratory syncytial virus’ (RSV) and ‘Epstein–Barr virus’ (EBV) [108].

Studies on infected shrimp, *Penaeus monodon*, have identified hemocyanin‐derived peptides that are able to neutralize WSSV (‘white spot syndrome virus’) and prevent replication [109]. In another study, it was found that WSSV particles preincubated with purified hemocyanin of *P. japonicus* prior to infection allowed a 10‐fold reduction of viral load in the gills compared with control shrimp that were not previously treated [110].

Only hemocyanin‐derived peptides from arthropods with antiviral activity were found only in crustaceans (arthropod viruses) [28, 109, 110], showing that rondonin may be the first antimicrobial peptide derived from spider’s hemocyanin with activity against human viruses.

According to the results obtained, it was proposed that the mechanism of action of this peptide probably involves internal components of the microorganisms. With these results of the RNA virus test, enveloped or not, we verified the protection of the cells, avoiding viral replication.

### Rondonin binding (DNA/RNA)

**Fig. 6:**
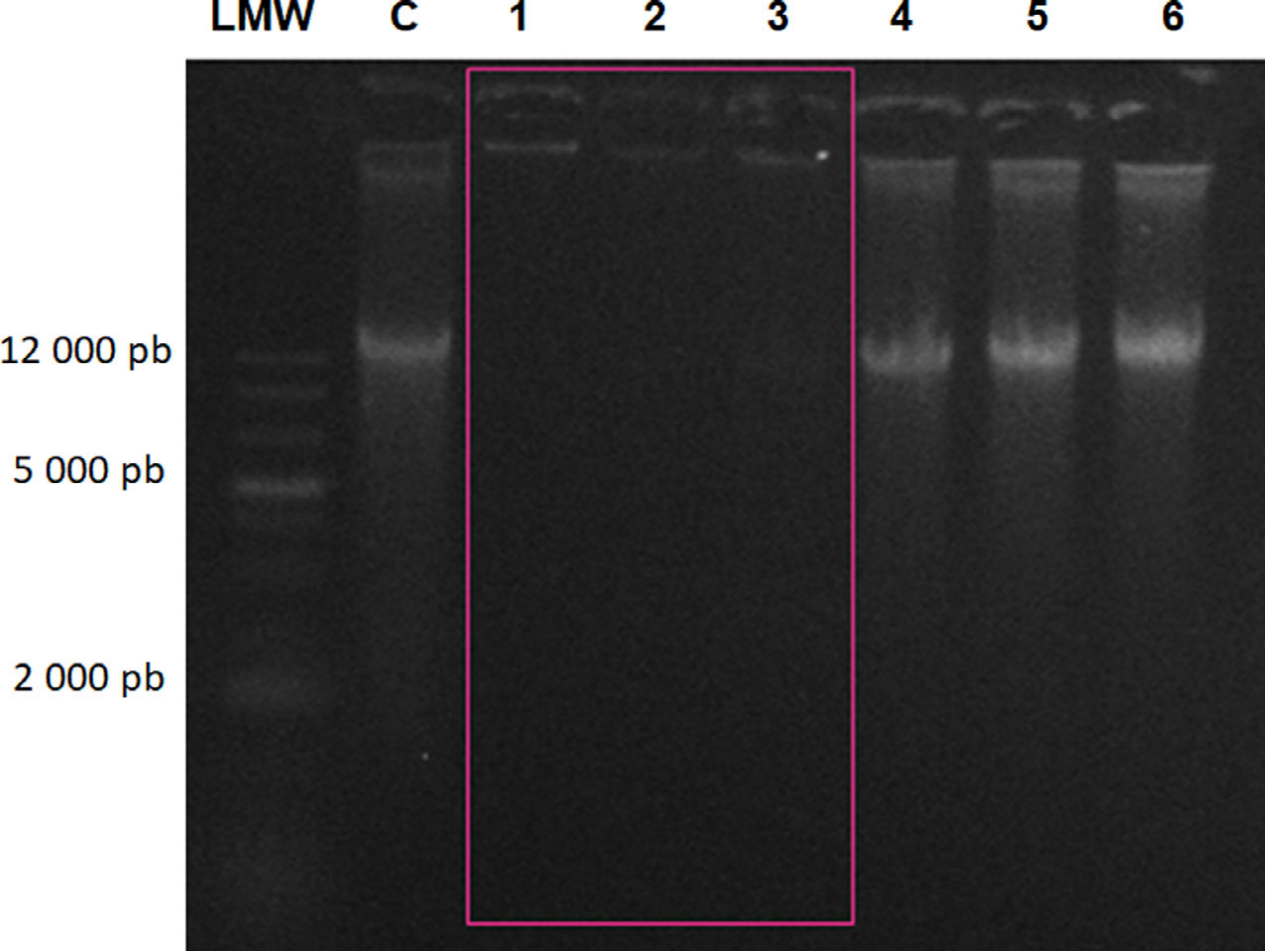
Agarose gel electrophoresis of 0.8% *Candida albicans* genomic DNA. The rondonin peptide at different concentrations (1–6) was incubated with 100 ng of genomic DNA from the yeast *C. albicans* for 10 min at room temperature. LMW—standard base pair marker, C—control, 1—12 μg, 2—6.2 μg, 3—4.9 μg, 4—3.7 μg, 5—2.4 μg, and 6—1.2 μg. The pink rectangle represents the binding of the peptide to the genetic material, thus preventing its migration in the gel. The test was performed in triplicate (*n* = 3).

**Fig. 7:**
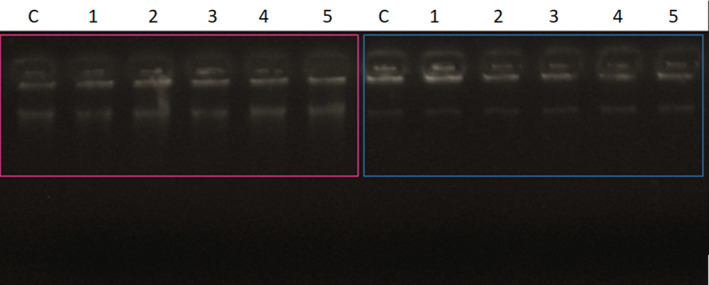
Electrophoresis in agarose gel 0.8% of genomic DNA from *Escherichia coli* (pink) and *Micrococcus luteus* (blue). The rondonin peptide at different concentrations (1–5) was incubated with 100 ng of *E. coli* bacterial genomic DNA for 10 min at room temperature. C—control, 1—6.2 μg, 2—4.9 μg, 3—3.7 μg, 4—2.4 μg, and 5—1.2 μg. The rondonin peptide at different concentrations (6–10) was incubated with 100 ng of genomic DNA from the *M. luteus* bacterium. C—control, 6—6.2 μg, 7—4.9 μg, 8—3.7 μg, 9—2.4 μg, and 10—1.2 μg. The test was performed in triplicate (*n* = 3).

Since that rondonin have antiviral activity and nonmembrane targets, its DNA/RNA‐binding ability was evaluated in a gel retardation assay against *C. albicans* MDM8, *E. coli* D31, and *M. luteus* A270. Peptides (0–12 µg) were mixed with a fixed amount (100 ng) of genomic DNA and total RNA (89 ng), incubated at room temperature for 5 min, and electrophoresed on a 0.8% agarose gel in TAE buffer (40 mm Tris pH 8.3, 20 mm acetic acid, and 1 mm EDTA). From 1.2 to 3.6 µg of peptide, the genomic DNA of *C. albicans* MDM8 was still able to migrate into the gel in the same way as noncomplexed DNA, whereas at 4.8 µg, almost all of the DNA remained at the origin. At higher concentrations of rondonin, complete blocked of the DNA was observed, showing that the DNA was aggregated by rondonin (Fig. 6). The same amount of peptide was tested against genomic DNA of *E. coli* and *M. luteus*, showing no retardation (Fig. 7). Similar patterns of migration were observed with Indolicidin [111].

**Fig. 8:**
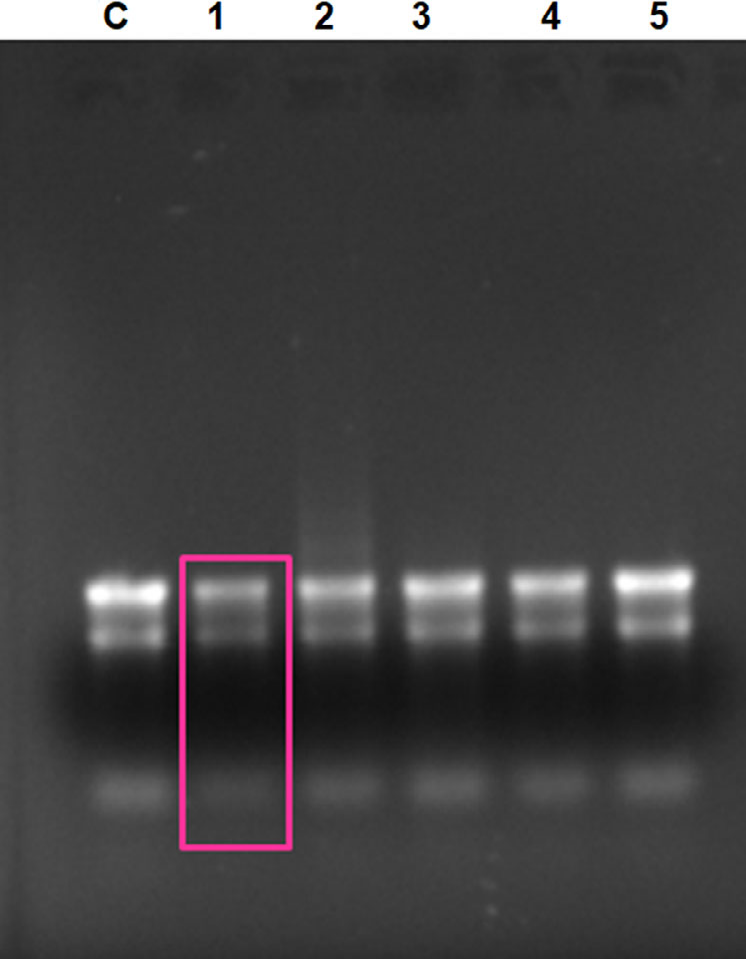
Agarose gel electrophoresis of 0.8% of total RNA from *Candida albicans*. The synthetic peptide rondonin at different concentrations (1–5) was incubated with 89 ng of total RNA (treated with DNase I for the removal of genomic DNA) from the yeast *C. albicans* for 10 min at room temperature. C—control (RNA only), 1—6.2 μg, 2—4.9 μg, 3—3.7 μg, 4—2.4 μg, and 5—1.2 μg. The pink rectangle represents the binding of the peptide to the genetic material, thus preventing its migration in the gel. The test was performed in triplicate (*n* = 3).

The RNA‐binding ability of rondonin was also evaluated by gel retardation on a 0.8% agarose gel (Fig. 8). The results show that rondonin has intrinsic DNA‐binding and low RNA‐binding ability, similar to what was seen for indolicidin, where low RNA binding occurs [111].

**Fig. 9:**
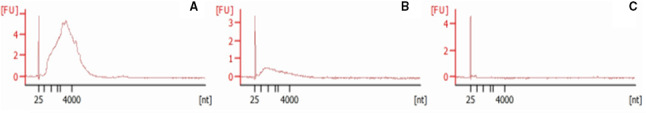
Capillary electrophoresis for mRNA evaluation of *Candida albicans* MDM8. (A) As a positive control, only mRNA (7.5 ng) was evaluated. (B) As a negative control, only the peptide was evaluated. (C) The synthetic peptide rondonin at a concentration of 6.2 μg was incubated with 7.5 ng mRNA from the yeast *C. albicans* for 10 min at room temperature and analyzed by capillary electrophoresis. (*n* = 1).

Rondonin (6 µg) was mixed with a fixed amount of mRNA (7.5 ng), and at this concentration, the peptide was able to completely inhibit mRNA (Fig. 9).

Previous studies have shown a similar result for the peptide indolicidin, which is able to bind to yeast plasmids and RNA. The concentration of the peptide used in our experiment was lower than that used with indolicidin; however, the amount necessary for the delay to occur can be smaller since the plasmid used is smaller than the genomic DNA [111]. The same was observed to the synthetics peptides KWKW‐NH2 (KW2), KWKWKW‐NH2 (KW3), KWKWKWKW‐NH2 (KW4), and KWKWKWKWKW‐NH2 (KW5) [71].

### Rondonin–FITC coupling confirmation

**Fig. 10:**
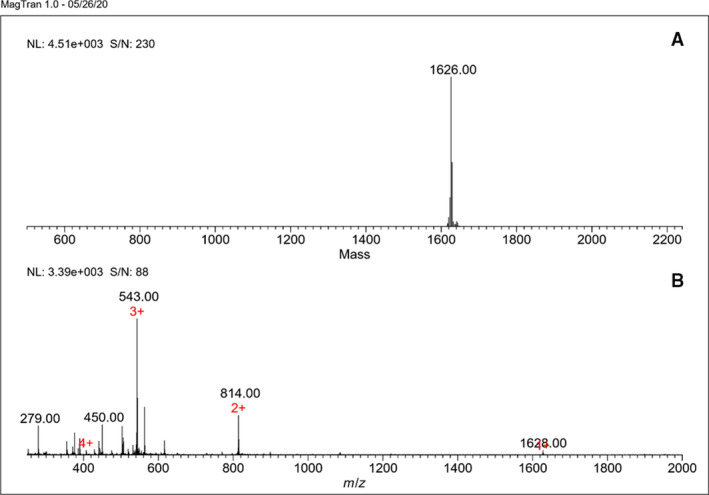
Determination of the molecular mass of *rondonin*–FITC obtained by the LC‐MS mass spectrometer. (A) Deconvolution of the ion profile resulting in a mass of 1626 Da. (B) Profile of ions obtained in the mass spectrometer used for deconvolution.

First, to verify the rondonin–FITC coupling, the complex was examined by mass spectrometry LC‐MS, and the conjugate was present on the FITC molecule for each rondonin sequence, confirming the coupling (Fig. 10).

#### Fluorescence analysis

Intracellular translocation or membrane attack of an AMP is the two important phenomena for candicidal targets. Some peptides require intracellular localization for toxic effects, such as Histatin 5 and P113 [112, 113], or they are membrane attacking peptides, such as the di‐18Hc peptide [114]. To determine whether rondonin could be translocated into the cytoplasm, its cellular localization was observed by confocal fluorescence microscopy using propidium iodide (PI) and FITC‐labeled rondonin (100 µm, F‐rondonin).

**Fig. 11:**
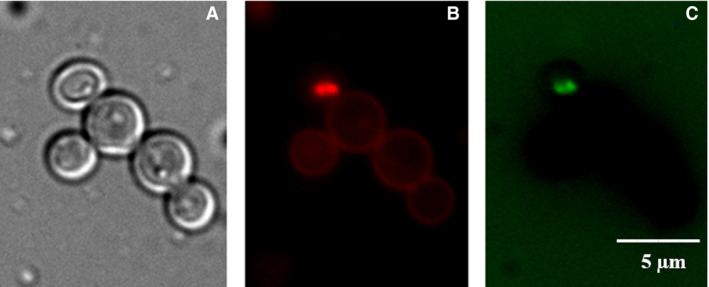
Fluorescence microscopy of *C. albicans* MDM8 cells incubated for 1 h at 37 °C and stained with PI (red) or *rondonin*–FITC (green). (A) Untreated control cells, PBS. (B) Cells treated with *rondonin*–FITC + PI. (C) Cells treated with *rondonin*–FITC. PI assay revealed the nucleus colors when treated with *rondonin*–FITC, and FITC‐stained cells showed the localization of peptide inside the cell. The test was performed in triplicate (*n* = 3). Scale bar = 5 µm.

As we can see in Fig. 11, the rondonin–FITC is located in the nucleus. We could see the PI revealed the nucleus in red color and rondonin–FITC in green. Overall, our results suggest that rondonin does not perturb the plasma membrane as part of its mechanism of action, but that nucleic acids in the nucleus of fungal cells might be the target site for rondonin that gives rise to its fungicidal action against *C. albicans*.

## Conclusion

The results presented here shed light on the mechanism of action of the AMP rondonin against *C. albicans*, as well as its antiviral property and synergism with another AMP (gomesin). The peptide presents higher antifungal activity at acidic pH, similar to those found in the vaginal environment, and, contrary to the mechanism of action of most AMPs, the peptide does not act at the membrane level. The nuclear localization and binding to genetic material suggested that rondonin antifungal activity involves the interaction with yeast DNA. These results in conjunction with its low cytotoxicity point to its potential therapeutic use for the treatment of infectious diseases, in particular candidiasis, either by itself or in combination with other antimicrobial compounds.

## Conflict of interest

The authors declare no conflict of interest.

## Author contributions

KCTR and PISRJ carried out the molecular laboratory work and sample preparation, participated in data analysis, and participated in the design of the study. KCTR carried out antimicrobial assays and checkboard assays and drafted the manuscript. KCTR and UCO carried out genetic material assays. RZM carried out antiviral assays. JCBJ and SS carried out membrane interaction assays. PISJ and JCBJ contributed to the revision of the manuscript. All authors gave final approval for publication and agreed to be held accountable for the work performed therein.

## Data Availability

The data that support the findings of this study are presented in the main manuscript of this article.
